# Downregulated Copper Homeostasis-Related Gene FOXO1 as a Novel Indicator for the Prognosis and Immune Response of Breast Cancer

**DOI:** 10.1155/2022/9140461

**Published:** 2022-06-28

**Authors:** Rong Zeng, Bi Peng, Emin Peng

**Affiliations:** ^1^General Surgery Department, Second Xiangya Hospital, Central South University, Changsha 410008, China; ^2^Xiangya International Medical Center, Xiangya Hospital, Central South University, Changsha 410008, China; ^3^National Clinical Research Center for Geriatric Disorders, Xiangya Hospital, Central South University, Changsha 410008, China

## Abstract

Copper (Cu) is one of the essential microelements for all living systems. Studies have illustrated the biological significance of Cu homeostasis in human cancers, including breast cancer (BRCA). Nevertheless, the detailed roles of Cu homeostasis in BRCA need to be further explored. Here, we identified a downregulated Cu homeostasis-related gene FOXO1 and investigated the potential functions of FOXO1 in BRCA through several bioinformation databases. The BRCA patients with high level of FOXO1 displayed favorable prognostic values. Subsequently, enrichment analysis of FOXO1 coexpressed genes revealed that the top three enriched KEGG pathways were spliceosome, oxidative phosphorylation, and ribosome. Immunoinfiltration analysis indicated that aberrantly expressed FOXO1 showed positive correlations with the subcellular infiltration of macrophages and neutrophils in BRCA. Moreover, FOXO1 expression was positively associated with multiple immune checkpoints, such as sialic acid-binding immunoglobulin-like lectin 15 (SIGLEC15), indoleamine 2,3-dioxygenase 1 (IDO1), programmed cell death 1 ligand 1 (PD-L1/CD274), hepatitis A virus cellular receptor 2 (HAVCR2), programmed cell death 1 (PDCD1), cytotoxic T lymphocyte antigen 4 (CTLA4), and programmed cell death 1 ligand 2 (PDCD1LG2). Overall, these findings would deepen our understanding of FOXO1 in BRCA prognosis and immunotherapy response, representing a promising therapeutic strategy for BRCA patients.

## 1. Introduction

Breast cancer (BRCA) remains a major problem affecting women's health and is the second leading cause of cancer-related deaths among women worldwide. About 2.2 million people were diagnosed with BRCA each year, and a third of them die from this disease [1]. In recent years, advanced and effective treatments have brought a steady decline in mortality from BRCA. However, the incidence of BRCA has risen, and the age of onset has become much younger [2]. Thus, a need for a novel indicator to improve outcomes of patients with BRCA is essential and urgent.

Copper (Cu) is an essential micronutrient participating in various life processes. Precise regulation of Cu homeostasis is the key point to maintain fundamental biological functions and prevent the occurrence of related disease. Cu homeostasis has been reported to be involved in cell proliferation, angiogenesis, and metastasis [3]. Meanwhile, several reports have demonstrated that Cu homeostasis and Cu-binding proteins were involved in many cancers, including BRCA, colorectal cancer, and lung cancer. Researchers have observed the increased Cu level in these cancer patients in comparison with healthy control groups [4]. Notably, serum Cu level has been proven to be implicated in the stage and progression of BRCA [5].

FOXO1, a winged-helix transcription factor, regulates a lot of physiological processes such as glucose homeostasis, apoptosis, autophagy, and cell cycle control. Additionally, FOXO1 promotes the expression of metal-containing antioxidant proteins including Cu-containing proteins and plays an important role in Cu homeostasis [6]. FOXO1 is also involved in pathological processes such as metabolic diseases and cancers, including liver disease [7] and BRCA [8]. However, few studies have addressed the regulatory roles of FOXO1 in the immune microenvironment in BRCA patients.

A comprehensive investigation was conducted to reveal the roles of Cu homeostasis-related genes in BRCA. This study mainly focused on the potential functions and mechanisms of Cu homeostasis-related gene, FOXO1, in BRCA, representing a promising prognostic and therapeutic target.

## 2. Methods

### 2.1. Data Acquisition

The data were obtained from the Gene Expression Omnibus (GEO) database according to the following conditions: (1) tumor type: BRCA, (2) species: Homo sapiens, and (3) analysis type: tumor vs. normal. We finally determined two GEO datasets (GSE10797 and GSE15852) [9, 10]. A Cu homeostasis-related gene dataset was retrieved from the MalaCards human disease database [11]. Then, gene expression profiles of two GEO datasets were downloaded to analyze differentially expressed genes. The *p* value was less than 0.01, and |log FC| was greater than 1. Next, a Venn diagram was exploited to identify the codifferentially expressed genes (co-DEGs) among two GEO datasets and the Cu homeostasis-related gene dataset.

### 2.2. Bioinformatics Analysis

DRUGSURV, an open-access web resource, is devoted to providing the statistical evidence of the drugs affecting patients' outcomes by analyzing survival information of cancer patients [12]. It was employed to explore the prognosis values of candidate co-DEGs in prognosis with BRCA. We assessed the expression levels of FOXO1 in tumor and control groups from GSE10797 and GSE15852. Transcriptional levels of FOXO1 in BRCA patients were further demonstrated in unpaired and paired samples from The Cancer Genome Atlas (TCGA) database. Then, TNMplot, a convenient tool for comparing gene expression profiles in normal, tumor, and metastatic tissues, was also applied to analyze the expression levels of FOXO1 [13]. In addition, TCGA-BRCA dataset was used to investigate the roles of FOXO1 in patients' clinical characteristics, such as stages, race, histological type, and progesterone receptor (PR) status.

The following work focused on coexpression analysis of FOXO1, which was realized by the LinkedOmics algorithm [14]. We obtained the 50 coexpressed genes positively and negatively related to FOXO1 though the LinkFinder module. The results were presented in the form of volcano plots and heat maps. Furthermore, gene set enrichment analysis (GSEA) was performed though the LinkInterpreter module. Kyoto Encyclopedia of Genes and Genomes (KEGG) pathways and Gene Ontology (GO) terms were also investigated.

The single-sample GSEA (ssGSEA) algorithm was performed to explore the associations between FOXO1 expression and tumor-infiltrating immune cells in BRCA. Subsequently, the Tumor IMmune Estimation Resource 2.0 (TIMER2.0) database [15] and Tumor and Immune System Interaction Database (TISIDB) database [16] were utilized to validate their associations. Finally, we investigated the relationships between FOXO1 expression with immunomodulators and chemokines using the TISIDB database. The main tools used in this research are summarized in Table 1.

## 3. Results

### 3.1. Determining the Differentially Expressed Genes

**Figure 1:**
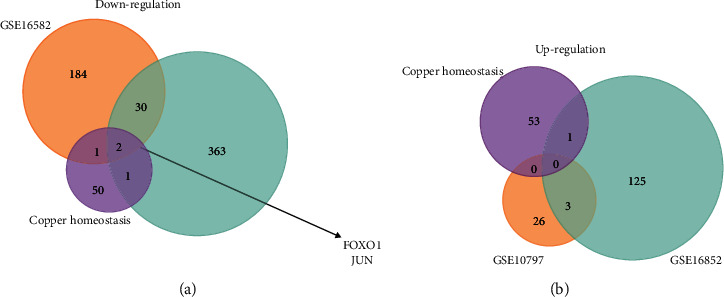
Venn analysis for determining co-DEGs. (a, b) Two downregulated co-DEGs, namely, FOXO1 and JUN, were determined though two GEO datasets and the Cu homeostasis-related gene dataset.

The expression profiles of two GEO datasets (GSE15852 and GSE10797) were exploited to screen differentially expressed genes between tumor tissues and normal tissues. The screen criteria were that *p* value was less than 0.01 and |log FC| was greater than 1. We found 217 downregulated genes and 129 upregulated genes in GSE15852 and 396 downregulated genes and 29 upregulated in GSE10797, respectively. Subsequently, Venn plots between two GEO datasets and Cu homeostasis-related gene dataset presented two downregulated co-DEGs, FOXO1 and JUN (Figures 1(a) and 1(b)). Unfortunately, there was no upregulated co-DEG associated with Cu homeostasis.

### 3.2. Identifying the Prognostic Values of Candidate Genes in Breast Cancer

**Figure 2:**
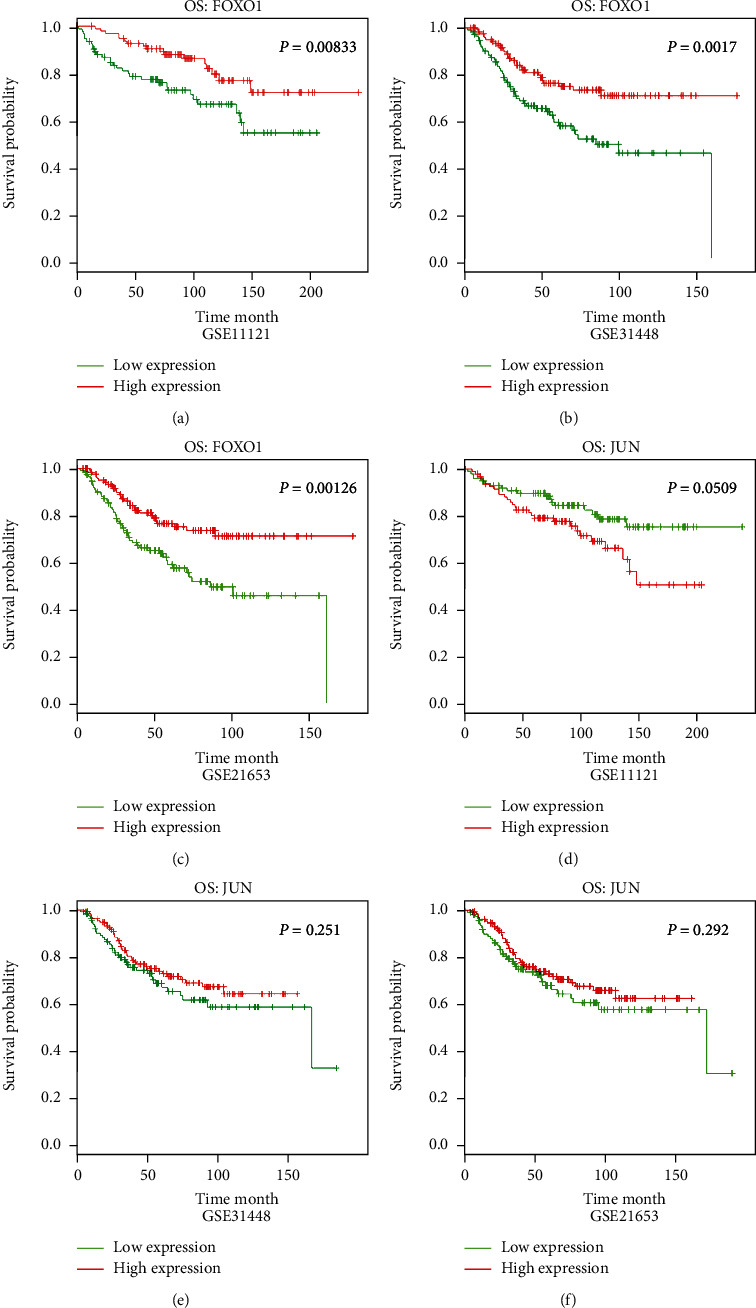
The prognosis values of FOXO1 in BRCA patients. (a–f) The prognostic values of FOXO1 and JUN were analyzed using the DRUGSURV platform in patients with BRCA from GSE11121, GSE31448, and GSE21653.

We utilized the DRUGSURV platform to obtain overall survival (OS) data of BRCA patients from GSE11121 [17], GSE31448 [18], and GSE21653 [19]. The effect of the expression levels of FOXO1 and JUN on the prognosis of patients with BRCA is shown in Figures 2(a)–2(f). Higher FOXO1 expression indicated good OS (*p* < 0.05). However, there was no significant correlation between the JUN expression and patients' OS (*p* > 0.05). These findings supported the underlying roles of FOXO1 in BRCA patients' prognosis. Hence, FOXO1 was the main focus for further research.

### 3.3. Validating the FOXO1 Expression and Exploring Its Clinical Significance

**Figure 3:**
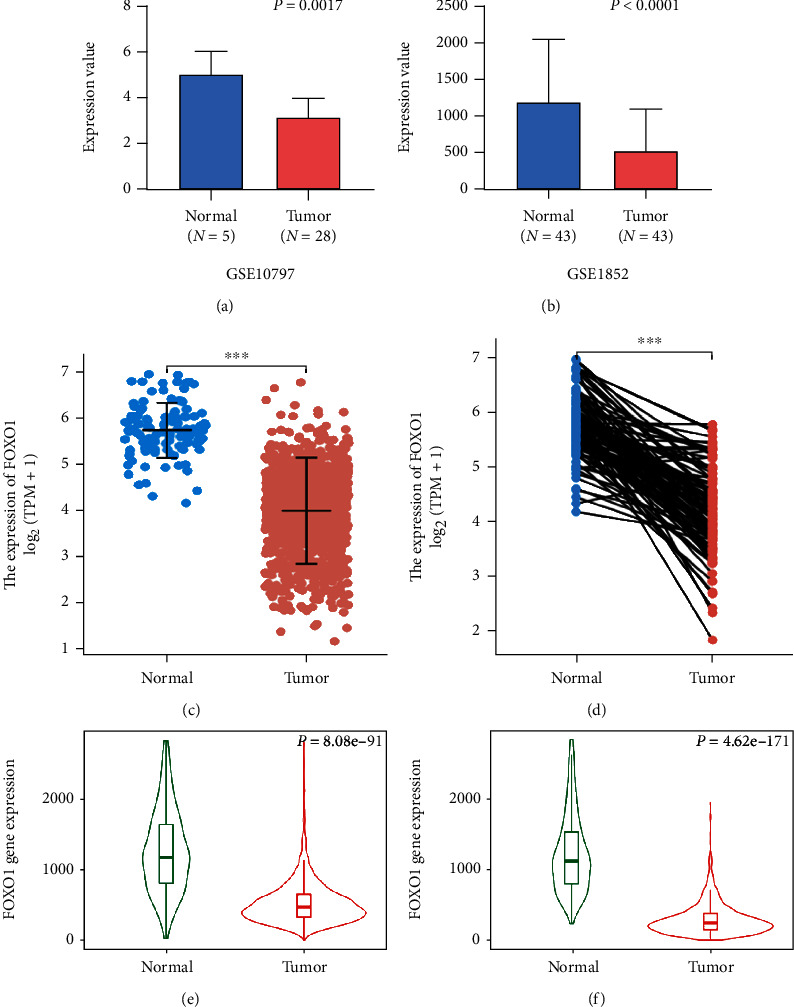
The differences of FOXO1 expression between tumor groups and normal control groups. (a–d) The downregulated FOXO1 expression was displayed in several datasets, including GSE10797 (a), GSE15852 (b), unpaired TCGA-BRCA (c), and paired TCGA-BRCA (d). (e, f) The downregulation of FOXO1 expression was validated using gene chip data (e) and RNA-seq data (f) in the TNMplot platform. ^∗∗∗^*p* < 0.001.

Firstly, we found the lower FOXO1 expression in tumor groups compared with normal controls in GSE15852 and GSE10797 (Figures 1(a), 3(a), and 3(b)). Then, integrating the data of TCGA-BRCA and Genotype-Tissue Expression (GTEx) suggested a consistent reduction of FOXO1 expression level in both unpaired and paired tissues (Figures 3(c) and 3(d)). What is more, TNMplot also implied downregulated expression levels of FOXO1 from gene chip data (Figure 3(e)) and RNA-seq data (Figure 3(f)).

The associations between FOXO1 expression and clinical characteristics of patients with BRCA were investigated in TCGA-BRCA patients. As shown in Table 2, the expression levels of FOXO1 were significantly associated with T stage (*p* = 0.004), race (*p* < 0.001), histological type (*p* < 0.001), PR status (*p* = 0.004), estrogen receptor (ER) status (*p* = 0.024), prediction analysis of microarray 50 (PAM50) (*p* < 0.001), menopause status (*p* = 0.013), and median age (*p* = 0.017). In contrast, N stage, M stage, pathologic stage, epidermal growth factor receptor 2 (HER2) status, and anatomic neoplasm subdivisions were not statistically significant. These results might provide a novel view for predicting the clinical progression of patients with BRCA based on FOXO1 expression.

### 3.4. Analyzing the Coexpression Network of FOXO1

**Figure 4:**
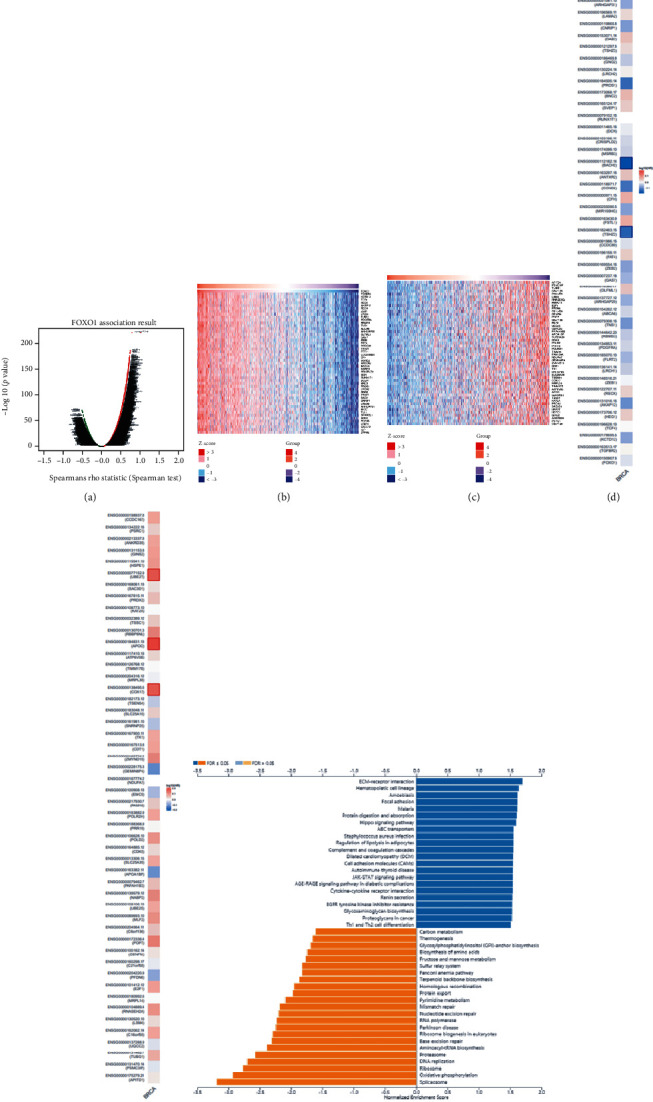
The coexpression network of FOXO1 in BRCA. (a) The coexpressed genes of FOXO1 were identified though the LinkedOmics platform. (b, c) The 50 genes having positive and negative correlations with FOXO1 were presented on heat maps. Red implied positively related genes, while blue indicated negatively related genes. (d, e) The top 50 genes positively and negatively connected with FOXO1 were displayed on the survival heat maps. (f) The KEGG pathway annotation of FOXO1 coexpressed genes in BRCA.

The coexpression model of FOXO1 in TCGA-BRCA was confirmed by the LinkedOmics database, which was conducive to exploring the underlying biological functions. Firstly, 9171 genes positively and 5976 genes negatively associated with FOXO1 are displayed in Figure 4(a). Two heat maps indicated the top 50 genes that had positive and negative correlations with FOXO1, respectively (Figures 4(b) and 4(c), Supplementary Tables 1 and 2). Furthermore, the prognostic significance of these genes was explored using GEPIA 2.0 [20]. An increased likelihood of being low-risk indicators in BRCA was displayed on 50 positive genes, and 15 of them had a protective hazard ratio (HR) (Figure 4(d)). On the contrary, an increased likelihood of becoming high-risk indicators in BRCA was presented on 50 negative genes, and 18 of them had an adverse HR (Figure 4(e)).

Studies on the potential biological functions of FOXO1 were carried out though GSEA. The results of the KEGG pathway are shown in Figure 4(f). Spliceosome, oxidative phosphorylation, and ribosome were the top three enriched KEGG pathways. Moreover, the results of GO terms suggested that mitochondrial respiratory chain complex assembly, respiratory chain, and rRNA binding were the main biological processes involved in FOXO1 coexpressed genes (Supplementary Figure S1A-C).

### 3.5. Investigating the Immune-Associated Roles of FOXO1 in Breast Cancer

**Figure 5:**
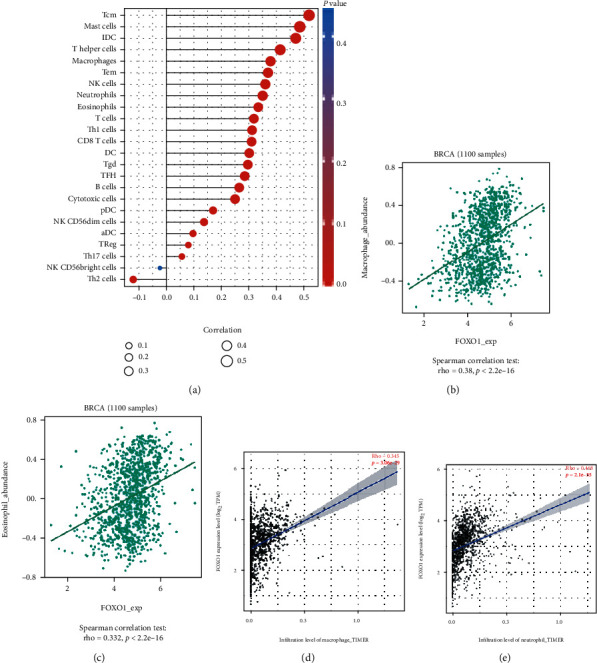
The regulatory roles of FOXO1 in immune infiltrating cells. (a) The relationships of FOXO1 expression and 24 kinds of immune-infiltrating cells. (b–e) The positive correlations between FOXO1 expression with macrophages and neutrophils were verified using TISIDB and TIMER2.0 platforms.

**Figure 6:**
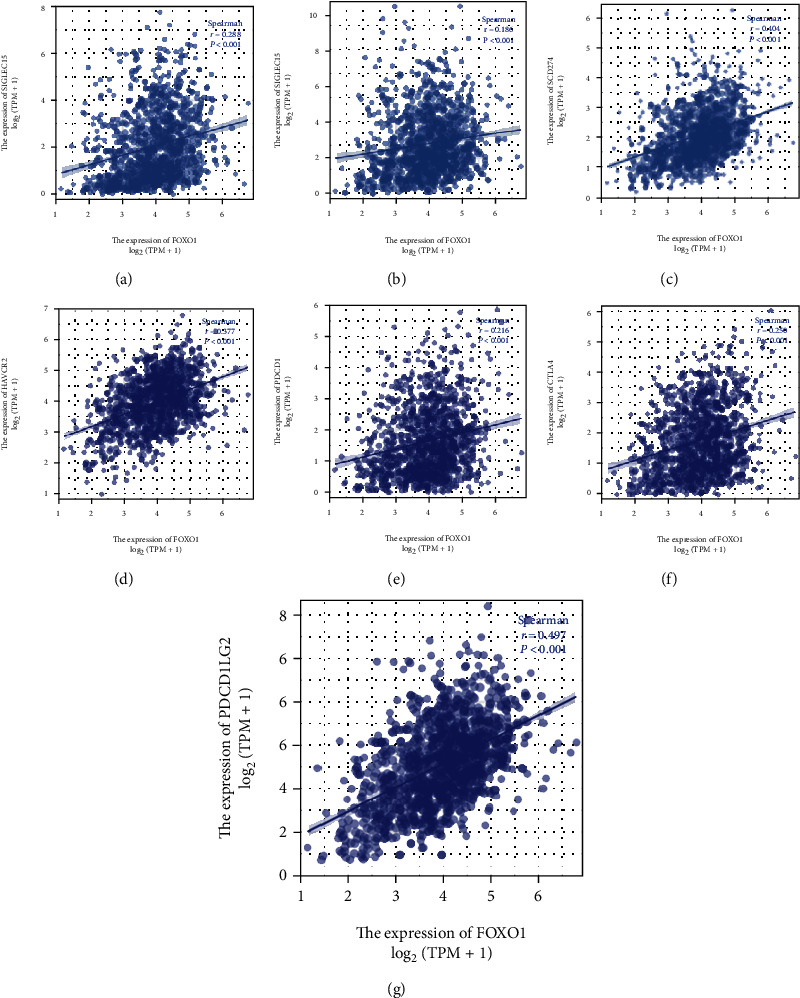
The relationships between FOXO1 expression and multiple immune checkpoints in BRCA patients. (a–g) We found the positive relationships between FOXO1 expression with multiple immune checkpoints, including SIGLEC15 (a), IDO1 (b), CD274 (c), HAVCR2 (d), PDCD1 (e), CTLA4 (f), and PDCD1LG2 (g).

We used the ssGSEA algorithm to investigate the correlations between FOXO1 and immune-infiltrating cells in TCGA-BRCA cohort. The results suggested that the top 10 immune-infiltrating cells having positive correlations with FOXO1 expression were central memory T cells (Tcm), mast cells, immature dendritic cells (iDC), T helper cells, macrophages, effector memory T cells (Tem), natural killers (NK) cells, neutrophils, eosinophils, and those having negative correlations with FOXO1 expression were Th2 cells (Figure 5(a)). We then validated these results using the TISIDB and TIMER 2.0 databases. As shown in Figures 5(b)–5(e), significantly positive associations with the expression of FOXO1 were also found in macrophages and neutrophils. Additionally, we revealed the positive relationships between FOXO1 expression with multiple immune checkpoints, including sialic acid-binding immunoglobulin-like lectin 15 (SIGLEC15), indoleamine 2,3-dioxygenase 1 (IDO1), programmed cell death 1 ligand 1 (PD-L1/CD274), hepatitis A virus cellular receptor 2 (HAVCR2), programmed cell death 1 (PDCD1), cytotoxic T lymphocyte antigen 4 (CTLA4), and programmed cell death 1 ligand 2 (PDCD1LG2) (Figures 6(a)–6(g)).

Next, other immune signatures related to the expression level of FOXO1 were investigated. Immunoinhibitors, immunostimulators, chemokines, and chemokine receptors were mainly included. The associations between FOXO1 expression and immunoinhibitors in patients with BRCA are displayed in Supplementary Figure S2A. The results showed that kinase insert domain receptor (KDR) (Spearman *r* = 0.414, *p* < 2.22*e* − 16), PDCD1LG2 (Spearman *r* = 0.406, *p* < 2.22*e* − 16), cell surface marker cluster of differentiation 96 (CD96) (Spearman *r* = 0.38, *p* < 2.22*e* − 16), and B and T Lymphocyte Attenuator (BTLA) (Spearman *r* = 0.37, *p* < 2.22*e* − 16) were the top four positively correlated molecules. Supplementary Figure S2B suggests the significant positive correlations between FOXO1 expression with the CXC chemokine ligand 12 (CXCL12) (Spearman *r* = 0.622, *p* < 2.22*e* − 16), 5′-Nucleotidase Ecto (NT5E) (Spearman *r* = 0.512, *p* < 2.22*e* − 16), ectonucleoside triphosphate diphosphohydrolase 1 (ENTPD1) (Spearman *r* = 0.496, *p* < 2.22*e* − 16), and CD40 ligand (CD40LG) (Spearman *r* = 0.38, *p* < 2.22*e* − 16). We then analyzed the relationships of FOXO1 expression and chemokines. The results indicated that the four significant molecules with the largest correlation coefficients were chemokine ligand12 (CXCL12) (Spearman *r* = 0.622, *p* < 2.22*e* − 16), C-C motif chemokine ligand 14 (CCL14) (Spearman *r* = 0.443, *p* < 2.22*e* − 16), C-C motif chemokine ligand 21 (CCL21) (Spearman *r* = 0.386, *p* < 2.22*e* − 16), and C-C motif chemokine ligand 22 (CCL22) (Spearman *r* = 0.375, *p* < 2.22*e* − 16) (Supplementary Figure S3A). Meanwhile, we also investigated the relationships of FOXO1 expression and chemokine receptors. As shown in Supplementary Figure S3B, C-C chemokine receptor type 2 (CCR2) (Spearman *r* = 0.412, *p* < 2.22*e* − 16), C-C chemokine receptor type 4 (CCR4) (Spearman *r* = 0.489, *p* < 2.22*e* − 16), C-C chemokine receptor type 6 (CCR6) (Spearman *r* = 0.328, *p* < 2.22*e* − 16), and C-C chemokine receptor type 5 (CCR5) (Spearman *r* = 0.311, *p* < 2.22*e* − 16) were the most significant chemokine receptors associated with FOXO1. Taken together, these findings suggested the roles of FOXO1 in the immune regulation and immunotherapy response in BRCA patients.

## 4. Discussion

In this research, we revealed the crucial role of Cu homeostasis-related gene FOXO1 in BRCA using multiple bioinformation databases. Firstly, two GEO datasets and the Cu homeostasis-related dataset were analyzed to screen the co-DEGs by Venn diagram. Then, the Kaplan-Meier plotter was used to evaluate the prognostic values of co-DEGs in BRCA patients. Moreover, the underlying roles of FOXO1 in the immune infiltration were also investigated. The results suggested the possibility of FOXO1 affecting BRCA progression though regulating the immune cell infiltration. Taken together, these findings could supply a new perspective for FOXO1 in the prognosis and treatment of BRCA patients.

Cu is a kind of necessary trace element to participate in the regulation of numerous physiological activities. Cu metabolism needs to be strictly controlled to prevent various diseases, including metabolic syndrome [21]. Absolutely, disordered Cu homeostasis can lead to a wide variety of cancers, including BRCA, colorectal cancer, and prostate cancer [22]. Several studies suggested that Cu was equipped to activate the mitogen-activated protein kinase (MAPK) pathway and facilitated tumorigenesis and cancer growth [23]. In addition, researchers observed that Cu levels were higher in the serum and tissues than those in healthy control groups when cancer patients were in advanced stages [24]. Likewise, previous studies implied a higher demand for Cu in cancer cells, which would be a breakthrough point to slow cancer progression [25]. Cu is considered a promising anticancer targeting agent [26]. There are two main types of Cu-targeting agents: Cu chelators and Cu ionophores. Cu chelators are aimed at combining with Cu and weakening its relative bioavailability. Its representative examples are Tetrathiomolybdate (TM), D-penicillamine (D-pen), and trientine [27]. In the past, Cu chelators were mainly used for the treatment of Wilson disease to bind accumulated Cu in the liver [28]. Afterwards, Cu chelators were found to play an important role in killing cancer cells [29]. Furthermore, Cu chelators may be a key to overcome platinum resistance, which has been proven by the MD Anderson Cancer Center. They used Cu chelators in combination with carboplatin to treat patients with platinum-resistant high-grade epithelial ovarian cancer and achieved a satisfactory result [30]. In contrast, Cu ionophores are designed to increase intracellular Cu levels by transferring Cu into cells. Its typical examples are clioquinol and disulfiram [24]. Mechanistically, these compounds can function by producing intracellular reactive oxygen species (ROS) and inhibiting proteasome activities, causing the apoptosis of cancer cells [31]. Overall, Cu homeostasis has a significant effect on various cancers and suggests new and potential anticancer approaches.

FOXO1, as a member of the forkhead box O (FOXO) family, is involved in the transcriptional regulation of genes, affecting biological and physiological processes [32]. It is well known for its roles in cell cycle, apoptosis, and cellular metabolism [33]. Aberrantly expressed FOXO1 has an impact on the development and prognosis of numerous tumors. For instance, a lack of FOXO1 expression is related to poor prognosis of patients with BRCA based on its function of inducing cell cycle arrest and apoptosis [34]. In addition, in urothelial carcinoma (UTUC), FOXO1 overexpression indicates worse outcomes due to its roles in accelerating growth and metastasis of tumor cells [35]. In recent years, the functions of FOXO1 in BRCA have been partially reported. Jeong et al. suggested that FOXO1 took part in the development of BRCA though regulating the nicotinamide phosphoribosyltransferase (Nampt) gene, which was involved in cell growth and angiogenesis [36]. Yu et al. thought that tribbles homologue 3 (TRIB3) overexpression activated the TRIB3-AKT1-FOXO1-SOX2 axis to support BRCA stemness, leading to the tumor reemergence and metastasis after chemotherapy and external radiation [37]. In this study, we found a reduced FOXO1 expression in BRCA, which might be associated with Cu homeostasis, indicating promising treatment strategies.

For the past few years, a growing number of studies have focused on tumor immune microenvironment (TIME), which has shown a significance in tumor immunosuppression, metastasis, and drug resistance [38]. More importantly, immunotherapy has become a new prospect in the treatment of tumors and has reaped encouraging effects on several solid tumors [39]. Generally, immune checkpoint blockades, containing programmed cell death receptor 1 (PD-1) and programmed cell death ligand 1 (PD-L1), have received clinical approval for the treatment of a wide variety of cancers such as melanoma, non-small-cell lung carcinoma (NSCLC), microsatellite instability-high (MSI-H), and mismatch repair deficiency (MMR-d) cancers [40]. BRCA has a high prevalence and generates a serious threat to women's health. The advent of immunotherapy has brought new hope to patients with BRCA. The currently main immunotherapies for BRCA include tumor-targeting antibodies, adoptive cell therapy, cancer vaccines, and immune checkpoint inhibitors [41]. In a phase II clinical trial performed by Mediratta et al.'s team, combination therapies of PD-1 and CTAL4 were employed to treat patients with triple-negative breast cancer, and 71% of them achieved excellent clinical outcomes [42]. Notably, cytotoxic T lymphocyte-associated antigen-4 (CTLA-4), PD-1 and PD-L1 have been approved by the US Food and Drug Administration (FDA) for the treatment of BRCA. In this researcher, the correlations between FOXO1 with immune infiltration were explored. The results suggested that macrophages and neutrophils had positive associations with FOXO1 expression. What is more, FOXO1 expression was significantly related to immunoinhibitors (KDR, PDCD1LG2, CD96, and BTLA), immunostimulators (CXCL12, NT5E, ENTPD1, and CD40LG), chemokines (CXCL12, CCL14, CCL21, and CCL22), and chemokine receptors (CCR2, CCR4, CCR6, and CCR5). Macrophages are ubiquitous and significant in human. They could be mainly classified as two subtypes, M1-like macrophages and M2-like macrophages [43]. The protease-activated receptor 2 (PAR2)/FOXO1 signal pathway stimulates polarization and inflammation of M1-like macrophages, which is causally linked to the occurrence of chronic disease [44]. FOXO1 can recruit M2-like macrophages to accelerate the progression of ESCC [45]. Moreover, FOXO1 affects regulation of neutrophils to phagocytize and kill bacteria [46]. The complementary pairing of KDR mRNA is mir-370-3p, which regulates the activity of the AKT/FOXO1 signaling pathway involved in intracranial aneurysm [47]. Increased activity of FOXO1 can enhance chemotactic reaction to CXCL12 [48]. CCL17 depended on CCR4 activation regulating the PI3K/AKT/FOXO1 signaling pathway to reduce neuronal inflammation and apoptosis after brain hemorrhage [49].

There were several deficiencies that need to be addressed. In this report, we mainly used comprehensive bioinformatics to demonstrate the downregulation of FOXO1 expression in BRCA. Moreover, we also demonstrated the potential roles of FOXO1 in the prognosis and immune response of BRCA patients. In the future, more in vivo and in vitro experiments will be conducted to clarify the biological function and underlying mechanisms of FOXO1 in BRCA pathogenesis, immune regulation, and therapeutic response.

## 5. Conclusion

In this research, we revealed that FOXO1, a Cu homeostasis-related gene, was significantly downregulated in BRCA. Highly expressed FOXO1 indicated favorable prognosis values in patients with BRCA. In addition, the expression level of FOXO1 was linked to immune infiltrating regulation and might affect patients' therapeutic response, such as immunotherapy. Consequently, these findings would shed light on FOXO1 as a novel promising prognostic and therapeutic target for BRCA patients.

## Figures and Tables

**Table 1:** The main bioinformatics tools used in this study.

Database	Samples	URL	References
DRUGSURV	Tissues	http://www.bioprofiling.de/GEO/DRUGSURV/index.html	[12]
TNMplot	Tissues	https://www.tnmplot.com/	[13]
LinkedOmics	Tissues	http://www.linkedomics.org/login.php	[14]
TIMER 2.0	—	http://timer.cistrome.org/	[15]
TISIDB	—	http://cis.hku.hk/TISIDB	[16]
GEPIA 2.0	—	http://gepia.cancer-pku.cn/	[20]

**Table 2:** The clinical characteristics for BRCA patients from TCGA database on the basis of FOXO1 expression levels.

Characteristic	Low expression of FOXO1	High expression of FOXO1	*p* values
*n*	541	542	
T stage, *n* (%)			0.004
T1	115 (10.6%)	162 (15%)	
T2	339 (31.4%)	290 (26.9%)	
T3	64 (5.9%)	75 (6.9%)	
T4	20 (1.9%)	15 (1.4%)	
N stage, *n* (%)			0.465
N0	266 (25%)	248 (23.3%)	
N1	177 (16.6%)	181 (17%)	
N2	51 (4.8%)	65 (6.1%)	
N3	36 (3.4%)	40 (3.8%)	
M stage, *n* (%)			0.595
M0	438 (47.5%)	464 (50.3%)	
M1	8 (0.9%)	12 (1.3%)	
Pathologic stage, *n* (%)			0.098
Stage I	78 (7.4%)	103 (9.7%)	
Stage II	328 (30.9%)	291 (27.5%)	
Stage III	116 (10.9%)	126 (11.9%)	
Stage IV	8 (0.8%)	10 (0.9%)	
Race, *n* (%)			<0.001
Asian	42 (4.2%)	18 (1.8%)	
Black or African American	121 (12.2%)	60 (6%)	
White	327 (32.9%)	426 (42.9%)	
Age, *n* (%)			0.152
≤60	288 (26.6%)	313 (28.9%)	
>60	253 (23.4%)	229 (21.1%)	
Histological type, *n* (%)			<0.001
Infiltrating ductal carcinoma	411 (42.1%)	361 (36.9%)	
Infiltrating lobular carcinoma	64 (6.6%)	141 (14.4%)	
PR status, *n* (%)			0.004
Negative	191 (18.5%)	151 (14.6%)	
Indeterminate	1 (0.1%)	3 (0.3%)	
Positive	316 (30.6%)	372 (36%)	
ER status, *n* (%)			0.024
Negative	133 (12.9%)	107 (10.3%)	
Indeterminate	0 (0%)	2 (0.2%)	
Positive	376 (36.3%)	417 (40.3%)	
HER2 status, *n* (%)			0.102
Negative	252 (34.7%)	306 (42.1%)	
Indeterminate	7 (1%)	5 (0.7%)	
Positive	85 (11.7%)	72 (9.9%)	
PAM50, *n* (%)			<0.001
Normal	10 (0.9%)	30 (2.8%)	
LumA	220 (20.3%)	342 (31.6%)	
LumB	145 (13.4%)	59 (5.4%)	
Her2	44 (4.1%)	38 (3.5%)	
Basal	122 (11.3%)	73 (6.7%)	
Menopause status, *n* (%)			0.013
Pre	100 (10.3%)	129 (13.3%)	
Peri	14 (1.4%)	26 (2.7%)	
Post	368 (37.9%)	335 (34.5%)	
Anatomic neoplasm subdivisions, *n* (%)			0.928
Left	280 (25.9%)	283 (26.1%)	
Right	261 (24.1%)	259 (23.9%)	
Age, median (IQR)	59 (49, 69)	57 (48, 65)	0.017

## Data Availability

The data were obtained from multiple public databases, whose links are as follows: (1) http://www.bioprofiling.de/GEO/DRUGSURV/index.html (DRUGSURV database), (2) https://www.tnmplot.com/ (TNMplot database), (3) http://www.linkedomics.org/login.php (LinkedOmics database), and (4) http://cis.hku.hk/TISIDB (TISIDB database).

## Supplementary Materials

Supplementary Materials Figure S1: the GO annotation of FOXO1 coexpressed genes in BRCA. (A) Biological process. (B) Cellular component. (C) Molecular functions. Figure S2: the immunomodulators related to FOXO1 attained from the TISIDB platform in BRCA. (A) The four most relevant immunoinhibitors with FOXO1 were shown on heat maps and scatter plots. (B) The four most relevant immunostimulators with FOXO1 were displayed on heat maps and scatter plots. Figure S3: the chemokines and receptors linked to FOXO1 attained from the TISIDB platform in BRCA. (A) The four most relevant chemokines with FOXO1 were shown on heat maps and scatter plots. (B) The four most relevant receptors with FOXO1 were displayed on heat maps and scatter plots. Table 1: the top 50 positively genes correlated with FOXO1. Table 2: the top 50 negatively genes correlated with FOXO1.
