# Supplementary material for: Downregulated Copper Homeostasis-Related Gene FOXO1 as a Novel Indicator for the Prognosis and Immune Response of Breast Cancer
Source: J Immunol Res. 2022 Jun 28;2022:9140461. doi: 10.1155/2022/9140461 (PMC9256448; doi:10.1155/2022/9140461)
Supplement: Supplementary Materials — Figure S1: the GO annotation of FOXO1 coexpressed genes in BRCA. (A) Biological process. (B) Cellular component. (C) Molecular functions. Figure S2: the immunomodulators related to FOXO1 attained from the TISIDB platform in BRCA. (A) The four most relevant immunoinhibitors with FOXO1 were shown on heat maps and scatter plots. (B) The four most relevant immunostimulators with FOXO1 were displayed on heat maps and scatter plots. Figure S3: the chemokines and receptors linked to FOXO1 attained from the TISIDB platform in BRCA. (A) The four most relevant chemokines with FOXO1 were shown on heat maps and scatter plots. (B) The four most relevant receptors with FOXO1 were displayed on heat maps and scatter plots. Table 1: the top 50 positively genes correlated with FOXO1. Table 2: the top 50 negatively genes correlated with FOXO1. [file 9140461.f1.zip › 9140461.f1/Supplementary Table 2 (1).docx]

Supplementary Table 2. The top 50 negatively genes correlated with FOXO1

| Name | Statistic | P-value | FDR (BH) |
| --- | --- | --- | --- |
| APITD1 | -0.50294899 | 3.89E-71 | 1.25E-69 |
| PSMC3IP | -0.49775712 | 1.72E-69 | 5.28E-68 |
| TUBG1 | -0.4965254 | 4.20E-69 | 1.27E-67 |
| C6orf125 | -0.48905633 | 8.57E-67 | 2.44E-65 |
| C16orf59 | -0.47380092 | 3.00E-62 | 7.29E-61 |
| LSM4 | -0.46481894 | 1.11E-59 | 2.49E-58 |
| RNASEH2A | -0.46397884 | 1.92E-59 | 4.25E-58 |
| MRPL14 | -0.46388759 | 2.04E-59 | 4.50E-58 |
| E2F1 | -0.46293591 | 3.76E-59 | 8.26E-58 |
| PFDN6 | -0.44668193 | 1.02E-54 | 1.93E-53 |
| C21orf58 | -0.44615256 | 1.41E-54 | 2.65E-53 |
| CENPM | -0.44469153 | 3.42E-54 | 6.36E-53 |
| POP7 | -0.44342578 | 7.36E-54 | 1.35E-52 |
| C6orf136 | -0.44288531 | 1.02E-53 | 1.87E-52 |
| MLF2 | -0.44146705 | 2.40E-53 | 4.35E-52 |
| UBE2S | -0.4411472 | 2.90E-53 | 5.24E-52 |
| OBFC2B | -0.4400106 | 5.73E-53 | 1.03E-51 |
| PAFAH1B3 | -0.43961298 | 7.27E-53 | 1.30E-51 |
| APOA1BP | -0.43931443 | 8.69E-53 | 1.55E-51 |
| SLC25A39 | -0.43885226 | 1.14E-52 | 2.03E-51 |
| CDK5 | -0.43692359 | 3.59E-52 | 6.27E-51 |
| POLD2 | -0.43458007 | 1.43E-51 | 2.44E-50 |
| PRR19 | -0.43153959 | 8.43E-51 | 1.41E-49 |
| POLR2H | -0.43126892 | 9.87E-51 | 1.65E-49 |
| TIMM16 | -0.43006357 | 1.98E-50 | 3.28E-49 |
| FAM158A | -0.43003578 | 2.02E-50 | 3.33E-49 |
| NDUFA7 | -0.42740536 | 9.15E-50 | 1.48E-48 |
| GEMIN8P4 | -0.42688583 | 1.23E-49 | 1.98E-48 |
| ZMYND19 | -0.42564365 | 2.50E-49 | 3.97E-48 |
| CDT1 | -0.42513868 | 3.34E-49 | 5.29E-48 |
| TK1 | -0.42465404 | 4.39E-49 | 6.92E-48 |
| SNRNP25 | -0.42254779 | 1.45E-48 | 2.25E-47 |
| SLC25A10 | -0.42199462 | 1.97E-48 | 3.06E-47 |
| TSEN54 | -0.42165086 | 2.40E-48 | 3.71E-47 |
| COX17 | -0.42144969 | 2.68E-48 | 4.14E-47 |
| MRPL38 | -0.42143064 | 2.71E-48 | 4.18E-47 |
| TIMM17B | -0.42138057 | 2.79E-48 | 4.30E-47 |
| ATP6V0B | -0.4208582 | 3.74E-48 | 5.75E-47 |
| APOO | -0.41954728 | 7.78E-48 | 1.19E-46 |
| C20orf151 | -0.41875749 | 1.21E-47 | 1.83E-46 |
| TSSC1 | -0.41767989 | 2.20E-47 | 3.30E-46 |
| KAT2A | -0.41745464 | 2.49E-47 | 3.73E-46 |
| PRDX2 | -0.41708394 | 3.06E-47 | 4.57E-46 |
| SAC3D1 | -0.41618708 | 5.02E-47 | 7.43E-46 |
| UBE2T | -0.41611547 | 5.22E-47 | 7.71E-46 |
| HSPE1 | -0.41537757 | 7.83E-47 | 1.15E-45 |
| GINS2 | -0.41383549 | 1.83E-46 | 2.67E-45 |
| ANKRD39 | -0.41369245 | 1.97E-46 | 2.88E-45 |
| PSRC1 | -0.41339062 | 2.33E-46 | 3.39E-45 |
| C6orf129 | -0.41299535 | 2.89E-46 | 4.19E-45 |
